# Smooth muscle relaxant activity of *Crocus sativus* (saffron) and its constituents: possible mechanisms

**Published:** 2015

**Authors:** Amin Mokhtari-Zaer, Mohammad Reza Khazdair, Mohammad Hossein Boskabady

**Affiliations:** 1 *Neurogenic Inflammation Research Centre and Department of Physiology, School of Medicine, Mashhad University of Medical Sciences, Mashhad,Iran*; 2 *Student Research Committee, Mashhad University of Medical Sciences, Mashhad, Iran*

**Keywords:** *Crocus sativus*, *Saffron*, *Crocin*, *Safranal*, *Smooth muscle*, *Relaxant effect*, *Possible mechanisms*

## Abstract

Saffron, *Crocus sativus* L. (*C. sativus*) is rich in carotenoids and used in traditional medicine for treatment of various conditions such as coughs, stomach disorders, amenorrhea, asthma and cardiovascular disorders. These therapeutic effects of the plant are suggested to be due to its relaxant effect on smooth muscles. The effect of *C. sativus* and its constituents on different smooth muscles and the underlying mechanisms have been studied. Several studies have shown the relaxant effects of *C. sativus* and its constituents including safranal, crocin, crocetin and kaempferol on blood vessels. In addition, it was reported that saffron stigma lowers systolic blood pressure. The present review highlights the relaxant effects of *C. sativus* and its constituents on various smooth muscles. The possible mechanisms of this relaxing effect including activation of ß2-adrenoceptors, inhibition of histamine H_1_ and muscarinic receptors and calcium channels and modulation of nitric oxide (NO) are also reviewed.

## Introduction

*Crocus* *sativus* L (*C.* *sativus*), commonly known as saffron, is a small perennial plant belonging to the family of Iridaceae which is cultivated in many countries with mild and dry climate specially in Iran (Abdullaev, 1993 ▶). It has gray-green hairy margins leaves and grows to about 30-35 cm long and has a funnel-shaped reddish purple flower with red stigmas. The stigmas are commonly used as a flavoring and yellow coloring additive in various foods such as bread, soups, sauces and rice (Kataria et al., 2011 ▶). 

Main constituents of saffron stigmas are crocin (responsible for its color), picrocrocin (responsible for its bitter taste) and safranal (responsible for its odor and aroma). It also contains more than 150 volatile aroma-yielding compounds and many non-volatile active components, many of which are carotenoids including zeaxanthin, lycopene and various alpha- and beta-carotenes (Srivastava et al., 2010 ▶). 

In folk medicine, *C. sativus* has been used as an antispasmodic, eupeptic, gingival sedative, anticatarrhal, sedative, carminative, diaphoretic, expectorant, stimulant, stomachic, aphrodisiac and emmenagogue medicine (Nemati et al., 2008 ▶). 

There also appears to be adequate evidence to support therapeutic effects of saffron and also for its use in alleviating several health disorders.

Recent studies showed pharmacological effects of saffron such as antioxidant (Hosseinzadeh et al., 2009 ▶; Soeda et al., 2007 ▶) antitumor (Abdullaev, 2002 ▶; Aung et al., 2007 ▶; Mousavi et al., 2011 ▶), memory enhancing (Abe and Saito, 2000 ▶; Ghadrdoost et al., 2011 ▶; Hosseinzadeh and Ziaei, 2006 ▶; Pitsikas et al., 2007 ▶), antidepressant and anxiolytic (Hosseinzadeh et al., 2003; Hosseinzadeh and Noraei, 2009 ▶), genoprotective (Hosseinzadeh and Sadeghnia, 2007 ▶; Premkumar et al., 2001 ▶), antitussive (Hosseinzadeh and Ghenaati, 2006 ▶), cardioprotective (Goyal et al., 2010 ▶; Imenshahidi et al., 2010 ▶; Xu et al., 2006 ▶; Zhang et al., 2009 ▶) and neuroprotective activities (Essa et al., 2012 ▶; Mehri et al., 2012 ▶).

Some of saffron therapeutic effects on stomach disorders, colic, dysmenorrhoea, asthma and cardiovascular disorders are suggested to be due to its relaxant effect on smooth muscles. A potent relaxant effect of the plant extract on tracheal smooth muscle was shown which was comparable to theophylline and greater than the effect of its constituent, safranal (Boskabady and Aslani, 2006 ▶). 

The effects of saffron petals extracts on blood pressure, hypotensive effect of aqueous extract of *C. sativus* and its constituents, safranal and crocin (Fatehi et al., 2003 ▶), effects of chronic and subchronic crocin treatment on hypertension (Imenshahidi et al., 2014 ▶; Razavi et al., 2013 ▶) and effect of chronic administration of saffron stigma aqueous extract on systolic blood pressure were shown (Imenshahidi et al., 2013 ▶). The protective effect of crocin on reperfusion-induced cardiac arrhythmias (Jahanbakhsh et al., 2012 ▶) as well as the lowering effect on heart rate and contractility was also documented (Boskabady et al., 2008 ▶). In addition, the effect of saffron on uterine contraction was also investigated (Chang et al., 1964 ▶). For *C. sativus* and its constituent safranal, a stimulatory effect on ß2-adrenoceptors (Nemati et al., 2008 ▶), an inhibitory effect on histamine H_1_ receptors (Boskabady et al., 2010 ▶) and a functional antagonistic effect on muscarinic receptors were demonstrated (Neamati and Boskabady, 2010 ▶). 

To prepare the present review, we explored the literature regarding the smooth muscle relaxant effect of *C. sativus* and its constituent as well as the possible underlying mechanisms. 

## Methods

The related data on this topic was obtained by searching for the terms "*Crocus sativus*", "saffron", "crocin", "crocetin", "safranal", "smooth muscles" and "relaxant effects" using ISI Web of Knowledge, Medline/Pubmed, Science direct, Scopus, Google Scholar, Embase, Biological Abstracts and Chemical Abstracts. 

**Constituents**

More than 150 compounds have been extracted from saffron stigmas, from which less than 50 chemicals have been completely characterized. Among fully characterized compounds, some are colored carotenoids e.g. crocetin and its glycosidic derivatives, crocins. The other components are colorless monoterpene aldehydes, volatile agents e.g. safranal, and bitter components, e.g. picrocrocin. The most important components are listed in a review article by Bathaie and Mousavi (Bathaie and Mousavi, 2010 ▶). 

Safranal (fat soluble) and pigments of the crocetin carotenoid (a natural di-carboxylic acid carotenoid precursor crocin) are bitter, but the most important cause of saffron bitterness is picrocrocin (Abdullaev, 1993 ▶). Color compounds of saffron are crocetin carotenoids and glycosidic forms of di-gentiobioside (crocin), gentiobioside, glycoside, gentio-glycoside and beta-crocetin di-glycoside (mono-methyl ester), gama-crocetin (di-methyl ester), alpha-carotene, beta-carotene, lycopene and zeaxanthin. Saffron lipophilic carotenoids are lycopene, alpha-and beta-carotene and zeaxanthin (Winterhalter and Straubinger, 2000 ▶; Tarantilis and Polissiou, 1997 ▶). Kampferol has also been found in alcoholic extract of saffron petals (Gregory et al., 2005 ▶). Flavonoids, especially lycopene, amino acids, proteins, starch, resins and other compounds have also been found in saffron (Assimopoulou et al., 2005 ▶). Moreover, saffron has trace amounts of thiamine and riboflavin (Alonso et al., 2001 ▶).

The main constituents of saffron are crocetin and its digentiobiosyl ester, crocin. Traces of non-glycosylated carotenoids unrelated to crocetin like β-carotene, lycopene and zea-xanthin are also present (Rios et al., 1996 ▶). Ethanolic extract of saffron has visible absorption peaks at 427 and 452 nm. When excited at 435 nm, saffron emits at 543 nm (Horobin et al., 2002 ▶). The water soluble extract of saffron stigmas is intensely colored; even one part of saffron in 100,000 parts of water yields a visibly yellow solution. The color components of saffron are diapocarotenoids, which are chemically similar to those in annatto.

Crocetin, another carotenoid that is isolated from saffron is one of the two principal chemicals responsible for saffron red color (Martin et al., 2002 ▶). Crocetin, a diapocarotenoid, is the result of crocin glycosides hydrolysis. Crocetin contains a carboxyl group at each end of the polyene chain; when ionized, it can function as an acid (anionic) dye for biological staining (Lillie, 1977 ▶). Crocetin in its free-acid form is insoluble in water and most organic solvents, except for pyridine and dimethylsulfoxide. Its anionic species are highly water soluble, so crocetin readily dissolves in dilute aqueous sodium hydroxide or other aqueous alkali solutions pH ≥ 9. Absorption maxima in pyridine are at 411, 436 and 464 nm. Crocetin constitutes approximately 0.3% of the total weight of the saffron stigma (Escribano et al., 1996 ▶; Dris and Jain, 2004 ▶). Crocetin is present mostly as its trans isomer. On the other hand, cis-crocetin and its glycosides are only minor components of saffron. 

Crocin (digentiobiosyl 8,8’-diapocarotene-8,8’-oate) belongs to a group of natural carotenoid commercially obtained from the dried stigma of saffron. It is the diester that is formed from the disaccharide gentiobiose and the dicarboxylic acid crocetin. 

Crocins account for almost 6–16% of saffron dry weight (based on its variety, cultivation environment and type of processing) (Gregory et al., 2005 ▶). Crocin (crocin 1) is a digentiobioside carotenoid which comprises the majority of crocins found in saffron. Due to its sugar parts, crocin could be easily dissolved in water, turning from a dark red dry powder to an orange aqueous solution making saffron a desirable coloring food additive (Melnyk et al., 2010 ▶). Its deeply red colored crystals have a melting point of 186^o^C. 

Crocin structure was elucidated by Karree (Karrer et al., 1933 ▶) though its presence was reported by Aschoff in the 19th centuary, as saffron main pigment(approx. 80%). Using water as the stationary phase and butanol as the mobile phase, crocin can be isolated from saffron extract in pure form and is directly crystallized.

Oral administration of single or multiple doses of crocin is not absorbed through the gastrointestinal tract and is largely excreted. It has been observed that orally administered crocins are hydrolyzed to crocetin before or during intestinal absorption, and absorbed crocetin is partly metabolized to mono- and diglucuronide conjugates (Asai et al., 2005 ▶). Crocetin does not tend to accumulate following administration of repeated oral doses and the intestinal tract serves as an important site for crocin hydrolysis (Xi et al., 2007 ▶).

**Relaxant effect of** ***C. sativus*** **and its constituents on different types of smooth muscles**

The relaxant effects of *C. sativus* extracts and constituents on various smooth muscles are reviewed in the following sections. 

**Vascular smooth muscle**

Imenshahidi et al. compared the hypotensive effect of saffron aqueous extract and its two active ingredients in rats. Based on the results, aqueous extract of saffron stigma, safranal and crocin decreased mean arterial blood pressure in a dose-dependent manner. The hypotensive effect of the extract is perhaps due to its relaxant effect on vascular smooth muscle. The results also suggested that safranal, the major constituent of the plant, contributes to the hypotensive activity (Imenshahidi et al., 2010 ▶). 

Fatehi et al. investigated the effects of *C. sativus* petals extract on blood pressure in anesthetized rats. Aqueous and ethanolic extracts of *C. sativus* petals reduced blood pressure in a dose-dependent manner. Administration of 50 mg/g of aqueous extract reduced blood pressure from 133.5 ± 3.9 to 117 ± 2.1 mmHg. This hypotensive effect could be either due to the effect of the *C. sativus* petals extracts on the heart itself or on total peripheral resistance via relaxation of vascular smooth muscle, or both. However, the results suggested that the effect of extracts on peripheral resistance seems to be more probable mechanism of this effect (Fatehi et al., 2003 ▶). 

It was also shown that chronic administration of aqueous extract of saffron stigma reduced DOCA-induced increase in mean systolic blood pressure (MSBP), but this hypotensive effect was not observed in normotensive rats. Data showed that antihypertensive effects of saffron did not last for a long period, so it could be postulated that long term blood pressure regulation systems were not affected by saffron (Imenshahidi et al., 2013 ▶). 

Aqueous-ethanolic extract of *C. sativus* also showed concentration-dependent inhibitory effect on heart rate and contractility comparable to the effect of diltiazem. The effect of plant extract on heart contractility could be due to its muscle relaxant effect (Boskabady et al., 2008 ▶).

It was also shown that crocetin (15, 30 mg/kg) dose-dependently improved endothelium-dependent relaxation (EDR) in response to acetylcholine (Ach) in high cholesterol diet (HCD)-fed rabbits. Also, in bovine aortic endothelial cells (BAECs), oxidized LDL (oxLDL) treatment decreased nitric oxide production and downregulated the activity and mRNA expression of endothelial nitric oxide synthase but these effects were inhibited by crocetin (0.1, 1, 10 mM) in a dose-dependent manner (Tang et al., 2006 ▶).

The effect of crocin (50 mg/kg) on improvement of the reduction of systolic blood pressure (SBP) and the increased heart rate (HR) induced by diazinon (DZN) in rats, was shown which could be due to the relaxant effect of crocin on muscle cells (Razavi et al. in press).

The vasodilator effects of quercetin and its metabolites on vessels resistance were also decumented (Xu et al., 2015 ▶). The relaxant effect of kaempferol, the other constituent of *C. sativus* was also examined in several studies. In one study, low concentration of kaempferol (10 μM) caused endothelium-dependent and endothelium-independent vascular relaxations (Xu et al., 2006 ▶). In addition, relaxant effect of kaempferol on smooth muscle cells of the porcine coronary artery was demonstrated (Xu et al., 2006 ▶). It has been also reported that quercetin and kaempferol relaxed rat aortic strips contraction induced by noradrenaline, KCI or phorbol 12-myris- tate,13-acetate (Duarte et al., 1993 ▶). 

**Tracheal smooth muscle**

In a study by Boskabady and Aslani (2006) ▶, on guinea-pig tracheal smooth muscle, it was shown that aqueous-ethanolic extract of saffron has a potent relaxant effect, which was comparable to theophylline. . Also, it was observed that there were positive correlations between increasing concentrations and the relaxant effects of the extract (Boskabady and Aslani, 2006 ▶). 

Safranal also showed concentration-dependent relaxant effect on tracheal smooth muscle. However, the relaxant effect of safranal was lower than that of the extract and theophylline (Boskabady and Aslani, 2006 ▶). These results indicated that relaxant effect of *C. sativus* is in part due to its constituent safranal. 

Long term oral administration of *C. sativus *extract (Bayrami et al., 2013 ▶) and its constituent safranal (Boskabady and Byrami, 2014 ▶) also reduced tracheal responsiveness to methacholine in sensitized guinea pigs. This effect could be due to relaxant effect of the extract and safranal on tracheal smooth muscle. The antitussive effect of the ethanolic extract of saffron and safranal was shown which could be due to their relaxant effect on airway smooth muscle (Hosseinzadeh and Ghenaati, 2006 ▶).

**Gastrointestinal and urogenital smooth muscle**

The effects of *C. sativus* petals extracts on isolated guinea-pig ileum induced by electrical field stimulation (EFS) was studied. In rat isolated ileum, contractile responses to EFS were decreased by the petals extracts. Contractions of ileum to EFS are mediated by both noradrenaline and ATP released as co-transmitters from sympathetic nerves (Hoyle and Burnstock, 1991 ▶). The results showed that ethanolic extract had a greater relaxant effect on EFS-induced contraction of guinea pig ileum than the aqueous extract (Fatehi et al., 2003 ▶).

The relaxant effect of saffron on uterine contraction was also demonstrated (Chang et al., 1964 ▶). The relaxant effect of *C. sativus* and its constituents on different types of smooth muscles was summarized in Table 1.

**Possible mechanisms underlying smooth muscle relaxant effect of** ***C. sativus***

*β2 – adrenoreceptors stimulatory effect*

The most possible mechanism for the relaxant effect of agents on tracheal smooth muscle is their stimulatory effect on β2-adrenergic receptors. The β2-adrenergic stimulatory effect of the plant and safranal was tested by performing cumulative concentration-response curves of isoprenaline-induced relaxation of pre-contracted isolated guinea pig tracheal smooth muscle. In this regard, aqueous ethanolic extracts from *C. sativus* (0.1 and 0.2 %), safranal (1.25 and 2.5 μg), 10 nM propranalol and saline were studied. The results showed clear leftward shifts in isoprenaline curves obtained in the presence of saffron extract and safranal compared to that of saline while propranolol caused rightward shift in isoprenaline response curve. The results indicated a relatively potent stimulatory effect for *C. sativus* extract and its constituent safranal on β_2_-adrenoreceptors (Nemati et al., 2008 ▶). Therefore, the results suggested that the major mechanism responsible for the relaxant effect of the plant and safranal is their stimulatory effect on β_2_-adrenoreceptors.

*Anti-cholinergic and anti-muscarinic effect of the plant*

The study of Nemati et al. (2010) ▶ demonstrated the functional antagonistic effect of *C. sativus* and safranal on muscarinic receptors on tracheal muscle of guinea pigs. Both the extract and safranal shifted methacholine concentration-response curve to the right. However, the shift was not parallel and the maximum response to methacholine in the presence of extract and safranal was not obtained. These results indicated a functional antagonistic effect of the plant and safranal on muscarinic receptors (Neamati and Boskabady, 2010 ▶).

**Table 1:** The relaxant effect of *C. sativus* and its constituents on different types of smooth muscle

**Type of smooth muscle (SM) **	**Solution**	**Effect**	**Reference**
**Vascular**	AE of saffron stigma, safranal and crocin	Decreased the mean arterial blood pressure of animals	Imenshahidi et al., 2010
	AE and EE of* C. Sativus* petals	Reduced the blood pressure in a dose-dependent manner	Fatehi et al., 2003
	AE of * C. Sativus stigma*	Reduced the enhanced of mean systolic blood pressure	Imenshahidi et al., 2013
	AE and EE of* C. Sativus*	Inhibitory effect on heart rate and contractility	Boskabady et al., 2008
	Crocetin, (15, 30 mg/kg)	Improved endothelium-dependent relaxation (EDR) in response to acetylcholine	Tang et al., 2006
	Crocin (50 mg/kg)	Crocin (50 mg/kg) and DZN in improvement of the reduction of SBP and the elevation of HR	Razavi et al. In press
**Trachea**	AE and EE of* C. Sativus*	Potent relaxing effect on the tracheal smooth muscle.	Boskabady et al., 2006
	Safranal	Has relaxant effect on tracheal smooth muscle	Boskabady and Aslani, 2006
	*C. Sativus*	Reduction effect on tracheal responsiveness to methacholine in animals	Bayrami et al., 2013
	Safranal	Reduction effect on tracheal responsiveness to methacholine in animals	Boskabady and Byrami, 2014
	AE of saffron and safranal	Antitussive effect was shown	Hosseinzadeh and Ghenaati, 2006
**Gastrointestinal**	Extract of* C. Sativus* petals	Induced relaxant effect in guinea -pig ileum	Fatehi et al., 2003
	Saffron	Relaxant effect on uterine contraction	Chang et al., 1964

Aqueous extracts (AE), ethanolic extracts (EE)

*Histaminic (H*_1_ *receptor) antagonistic activity*

The effect of three concentrations of aqueous-ethanolic extracts of *C. sativus* (0.025 %, 0.05 % and 0.1 %) on histamine (H_1_) receptors was evaluated in guinea pig tracheal smooth muscles. Concentration-response curve for histamine was obtained in the presence of saline, saffron extract and chlorpheniramine. The extract caused parallel rightward shift in histamine concentration-response curve similar to the effect of chlorpheniramine and the maximum response to histamine was obtained in the presence of the extract. These results showed an inhibitory effect of *C. sativus* (competitive antagonistic effect) on histamine H_1_ receptors which could be related to the relaxant effect of the plant on tracheal smooth muscle (Boskabady et al., 2010 ▶). 

The effect of safranal (0.63, 1.25 and 2.5 μg/ml) on histamine (H_1_) receptors in guinea pig tracheal smooth muscle was also studied using similar method as described above. The results showed that safranal also caused rightward shift in histamine–response curves, obtaining the maximum responses to histamine and greater EC_50_ (effective concentration of histamine causing 50% of maximum response). It was concluded that safranal possibly acts as a histamine H_1_ receptors competitive antagonist (Boskabady et al., 2011 ▶)**.**

*Calcium channel antagonistic effects*

Crocin could inhibit Ca^2+^ influx and release of intracellular Ca^2+^ stores in the endoplasmic reticulum in bovine aortic smooth muscle cells (He et al., 2004 ▶). It was also shown that reduction of intracellular Ca^2+^ release may contribute to relaxation of the corpus cavernosum, leading to erection (Williams et al., 2005 ▶). Coronary and other diseases in cardiac or brain blood vessels are considered to be due to the excessive influx of Ca^2+^ into cytoplasm. So, Ca^2+ ^channels blockers are of therapeutic value in treatment of these diseases. The effect of *C. sativus* on Ca^2+^ influx in isolated rat aortas was investigated using 45Ca as a radioactive tracer, and their calcium antagonistic effects were evaluated. Ca^2+ ^uptake in isolated rat aorta rings in normal physiological status was not markedly altered by these drugs, whereas Ca^2+^ influxe induced by norepinephrine 1.2 µmol/L and KCl 100 mmol/L were significantly inhibited by *C. sativus *in a concentration-dependent manner. 

The results showed that Ca^2+^ influx through receptor-operated Ca^2+^ channels and potential-dependent Ca^2+^ channels can be blocked by the plant (Liu et al., 2005 ▶). It is conceivable that the hypotensive effect of saffron in chronic treatment is related to its inhibitory effect on smooth muscles via blocking calcium channel or inhibiting sarcoplasmic reticulum Ca^2+^ release into the cytosol (Boskabady and Aslani, 2006 ▶).

Crocin also concentration-dependently inhibited the total cholesterol (TC) and Cholesteryl ester (CE) elevation induced by Ox-LDL. The results indicated that crocin could inhibit the increased intracellular [Ca^2+^] in smooth muscle cell (He et al., 2005 ▶).

Concerning the underlying mechanisms of the vasodilatory effect, protein kinase C inhibition and decrease in Ca^2+^ uptake were also investigated (Duarte et al., 1993 ▶). 

 It has been reported that crocetin decreased protein kinase C (PKC) activity in the membrane fraction, which led to reduced blood pressure by inhibition of proliferation in vascular smooth muscle cells (Cheng-Hua et al., 2010 ▶).

*Endothelium-dependent relaxation (EDR) effect*

The mechanisms by which crocetin causes vasorelaxation might be attributed to the vessel endothelial instead of direct effect on vessel smooth muscle. It was shown that crocetin, upregulated the eNOS mRNA expressions in both in vivo and in vitro studies which was in accordance with its action on eNOS activity and NO production. The NO formed in the endothelial cell is catalyzed by eNOS, crosses the plasma membrane and diffuses into the adjacent smooth muscle cells, where it binds and stimulates guanylyl cyclase, the enzyme that synthesizes cGMP. Cyclic GMP leads to a decrease in cytosolic Ca^2+^ concentration, which causes relaxation of the muscle cell and dilation of the blood vessel (Tang et al., 2006 ▶). 

Smooth muscle cells relaxations of the porcine coronary artery by endothelium-derived and exogenous NO due to endothelium-dependent hyperpolarization was shown. These results showed the involvement of activation of large-conductance calcium-activated potassium channel K_Ca_1.1 channels and NO in vascular smooth muscle relaxant effect (Xu et al., 2005 ▶). However, further studies should investigate this mechanism to justify the relaxant effect of *C. sativus* and its constituents.

*Increasing intracellular cAMP*

It was shown that rat uterine smooth muscle relaxation following KCl-induced tonic contraction was antagonized by Rp-cAMPS which indicates involvement of increased intracellular cAMP concentration in relaxing uterine smooth muscle (Revuelta et al., 2000 ▶). This mechanism should also be examined for *C. sativus* and its constituents in further experiments.

The possible mechanisms of the relaxant effect of *C. sativus* and its constituents were summarized in Table 2.

**Table 2:** Possible mechanisms of the relaxant effect of *C. sativus* and its constituents

**Possible mechanisms**	**Solution**	**Type of muscle**	**Reference**
**Stimulatory effect on β2-adrenoceptors**	AE extract of* C. Sativus* (0.1 and 0.2 g%) and safranal	Trachea	(Nemati et al., 2008)
**Anticholinergic and anti-muscarinic effect**	AE extract of* C. Sativus* and safranal	Trachea	(Neamati and Boskabady, 2010)
**Histaminic antagonistic activity**	AE extract of* C. Sativus* (0.025 g%, 0.05 g% and 0.1 g%)	Trachea	( Boskabady et al., 2010
	Safranal (0.63, 1.25 and 2.5 μg/ml)	Trachea	Boskabady et al., 2011
**Ca** ^2+^ ** channel blocking in aortic smooth muscle cells**	Crocin	Vascular	He et al., 2004
**Ca** ^2+^ ** channel blocking in corpus cavernosum leading to erection**	Crocin	Vascular	Williams et al., 2005
**Protein kinase C inhibition or decreased Ca ** ^2+^ ** uptake**	Kaempferol	Vascular	Duarte et al., 1993
**Protein kinase C inhibition ** **in the membrane fraction**	Crocetin	Vascular	Cheng-Hua et al., 2010
**Upregulation of eNOS mRNA expressions and NO production**	Crocetin	Vascular	(Tang et al., 2006
**Activation of large-conductance calcium-activated potassium channel k**_ca_ **1.1 channels and NO**	Kaempferol	Vascular	Xu et al., 2015
**Increase in intracellular cAMP**	Kaempferol	Vascular	Revuelta et al., 2000

## Conclusion

In conclusion, the relaxant effects of *C. sativus* and some of its constituents on vascular, trachea, gastrointestinal and urogenital smooth muscles were discussed in the present review article. The possible mechanisms of the relaxant effect of the plant and its constituents on smooth muscle including β_2_-adrenoreceptors stimulation, histamine (H_1_) receptor inhibition and calcium channel blocking were also reviewed. However, the point that exactly which chemical(s) through what mechanism(s) of action causes this effect as well as its clinical applications should be investigated in further studies.
